# Relationship between ventilation heterogeneity and exercise intolerance in adults with sickle cell anemia

**DOI:** 10.1590/1414-431X20176512

**Published:** 2017-07-17

**Authors:** A.J. Lopes, C.L. Marinho, U.D. Alves, C.E.A. Gonçalves, P.O. Silva, E.C. Botelho, R. Bedirian, A.R. Soares, M.C.P. Maioli

**Affiliations:** 1 Programa de Pós-Graduação em Ciências da Reabilitação, Centro Universitário Augusto Motta, Rio de Janeiro, RJ, Brasil; 2 Programa de Pós-Graduação em Ciências Médicas, Faculdade de Ciências Médicas, Universidade do Estado do Rio de Janeiro, Rio de Janeiro, RJ, Brasil; 3 Programa de Pós-Graduação em Fisiopatologia Clínica e Experimental, Universidade do Estado do Rio de Janeiro, Rio de Janeiro, RJ, Brasil; 4 Disciplina de Clínica Médica, Faculdade de Ciências Médicas, Universidade do Estado do Rio de Janeiro, Rio de Janeiro, RJ, Brasil; 5 Disciplina de Hematologia e Hemoterapia, Faculdade de Ciências Médicas, Universidade do Estado do Rio de Janeiro, Rio de Janeiro, RJ, Brasil

**Keywords:** Sickle cell anemia, Respiratory mechanics, Respiratory function tests, Ventilation, Exercise

## Abstract

Sickle cell anemia (SCA) causes dysfunction of multiple organs, with pulmonary involvement as a major cause of mortality. Recently, there has been growing interest in the nitrogen single-breath washout (N_2_SBW) test, which is able to detect ventilation heterogeneity and small airway disease when the results of other pulmonary function tests (PFTs) are still normal. Thus, the objectives of the present study were to assess the heterogeneity in the ventilation distribution in adults with SCA and to determine the association between the ventilation distribution and the clinical, cardiovascular, and radiological findings. This cross-sectional study included 38 adults with SCA who underwent PFTs, echocardiography, computed tomography (CT), and 6-min walk test. To evaluate the ventilation heterogeneity, the patients were categorized according to the phase III slope of the N_2_SBW (SIII_N2_). Compared with adults with lower SIII_N2_ values, adults with higher SIII_N2_ values showed lower hemoglobin levels (P=0.048), a history of acute chest syndrome (P=0.001), an elevated tricuspid regurgitation velocity (P=0.039), predominance of a reticular pattern in the CT (P=0.002), a shorter 6-min walking distance (6MWD) (P=0.002), and lower peripheral oxygen saturation (SpO_2_) after exercise (P=0.03). SIII_N2_ values correlated significantly with hemoglobin (r_s_=-0.344; P=0.034), forced vital capacity (r_s_=-0.671; P<0.0001), diffusing capacity for carbon monoxide (r_s_=-0.376; P=0.019), 6MWD (r_s_=-0.554; P=0.0003), and SpO_2_ after exercise (P=0.040). Heterogeneity in the ventilation distribution is one of the most common pulmonary dysfunctions in adults with SCA. Moreover, relationships exist between ventilation heterogeneity, worsening of pulmonary structural damage, and reduced tolerance for exercise.

## Introduction

Sickle cell anemia (SCA) is a disease with systemic repercussions. Multiple organ dysfunction occurs due to inflammation, abnormal blood rheology, vaso-occlusion, and endothelial dysfunction (1,2). The polymerization of hemoglobin S (Hb S) within the cell is the central event that occurs in the pathophysiology of SCA; however, there is increasing evidence that nitric oxide (NO) sequestration by cell-free Hb and impaired bioavailability of NO during intravascular hemolysis also play important roles in SCA pathophysiology (1,2). Respiratory system involvement accounts for approximately 30% of all deaths in patients with SCA and mainly involves acute chest syndrome (ACS) and pulmonary hypertension (PH) (1–4). ACS is one of the most important events in patients with SCA and is a manifestation of the vaso-occlusive phenomenon that can progress to acute respiratory distress syndrome, respiratory failure, and death (3,5). PH has a prevalence of approximately 10%, and its pathophysiology involves vaso-occlusive pulmonary phenomena, chronic hypoxia, pulmonary fibrosis, hemolysis, asplenia, iron overload, and endothelial dysfunction due to resistance to nitric oxide (6,7).

ACS, recurrent pulmonary infections, and obstructive airway phenomena contribute to a progressive deterioration of lung function throughout the lives of SCA patients (8). In this scenario, abnormal pulmonary function tests (PFTs) are one of the first objective signs of chronic lung disease in SCA patients and may be useful for the management of these patients (9). However, the correlations of PFT parameters with clinical, cardiovascular, and radiological findings have not been strong (4,8,10,11). Recently, interest in the nitrogen single-breath washout (N_2_SBW) test has been increasing because this test is a simple and non-invasive tool that is capable of detecting heterogeneity in ventilation distribution and small airway disease when other PFTs still present parameters within normal ranges (12). Additionally, the N_2_SBW test has shown strong correlations with several measurements used in clinical practice for some conditions, including chronic obstructive pulmonary disease and asthma (13,14). However, to the best of our knowledge, no study has evaluated the contribution of the N_2_SBW test to the detection of pulmonary function abnormalities in patients with SCA.

The pathophysiological contributors to the reduction of the functional capacity to exercise in individuals with SCA are not well understood (15). Some possible factors have been noted, including reduction of the oxygen transport capacity by Hb, pulmonary dysfunction resulting from repeated ACS episodes, cardiovascular abnormalities, osteoarticular alterations, generalized muscular weakness, and physical deconditioning (4,5,16–18). However, because deterioration of lung function is common in adults with SCA (8,9), it is of clinical interest to determine the impact of poor ventilation distribution on exercise performance in these patients. In this context, the N_2_SBW test may be a potential tool to predict functional capacity in adults with SCA.

Several factors may be involved in the poor ventilation distribution in SCA patients because the lungs are prone to constant episodes of vaso-occlusion, alveolar wall necrosis, and loss of lung units (8,10). In this scenario, the N_2_SBW test may play an important role because it may reflect the structural abnormalities that occur in SCA. Thus, the objectives of the present study were to assess the heterogeneity in the ventilation distribution in adults with SCA and to determine the association between the ventilation distribution and clinical and radiological findings, cardiovascular functions, and functional capacity in these patients.

## Patients and Methods

### Patients

Between July 2016 and January 2017, a cross-sectional study was performed to evaluate 47 adults with SCA regularly seen at the Hospital Universitário Pedro Ernesto, Universidade do Estado do Rio de Janeiro (Brazil). The following exclusion criteria were used: a history of ACS or vaso-occlusive crisis (VOC) in the last 4 weeks, a report of having received a blood transfusion in the last 3 months, a history of upper respiratory tract infection in the last 3 weeks, a history of smoking >10 packs per year, a report of previous pleuropulmonary disease not related to ACS, and any physical impairment that would impair the performance of the 6-min walk test (6MWT). The project was approved by the Research Ethics Committee of the Universidade do Estado do Rio de Janeiro (No. 13410613.5.0000.5259). All individuals signed the consent form.

### Procedures

All procedures were performed within 1 month. The PFTs were performed on Collins Plus Pulmonary Function Testing Systems (Warren E. Collins, Inc., USA) and consisted of the following tests: spirometry; body plethysmography; diffusion capacity for carbon monoxide (DLco); and respiratory muscle strength. All examinations followed the recommendations of the American Thoracic Society (19). The DLco values were corrected for the serum Hb values using the Brazilian reference values (20–23). In the present study, restrictive or obstructive patterns were defined when the total lung capacity (TLC) or the forced expiratory volume in 1 s/forced vital capacity (FEV_1_/FVC) were below the lower limit of normality.

We performed the N_2_SBW test on HDpft 3000 equipment (nSpire Health, Inc., USA) following the standard recommendations (12). Two parameters derived from the N_2_SBW test are reported as percentages of the predicted value (24), as follows: the phase III slope of the N_2_SBW (SIII_N2_), which is the change in the N_2_ fraction between 25–75% of the expired volume and is indicative of a poor ventilation distribution, and the closing volume/vital capacity (CV/VC), which is the portion of the VC that is expired after the beginning of airway closure and therefore served as a marker of small airway disease (12–14).

Transthoracic Doppler echocardiography was performed in an iE33 system (Philips Medical Systems, USA) following standard procedures (25). The images were stored on digital media for later interpretation. A tricuspid regurgitation velocity (TRV) value ≥2.5 m/s was considered high (25).

Computed tomography (CT) was performed on a helical CT with 64 channels (Brilliance 40; Philips Medical Systems). The current in the RX ampoule was 458 mA, and the voltage was 120 kV. Each acquisition consisted of cross-sectional cuts with a 2-mm thickness with a 1-mm distance between sections; the acquisitions were obtained during inspiratory and expiratory apnea without gantry inclination. The images are represented by an array of 512×512 columns. Each CT scan was classified into 3 categories, as follows: normal, predominance of a ground-glass opacification (GGO) pattern, or predominance of a reticular pattern (26). Two radiologists blind to the clinical and functional data independently assessed the CT findings. In case of divergence, a consensus was reached between the 2 radiologists to determine the final category.

The 6MWT was performed in a 30-m corridor, and the patients were familiarized with the procedure prior to testing. Peripheral oxygen saturation (SpO_2_) was measured before and after the end of the test. The step-by-step approach to the 6MWT was recommended by the American Thoracic Society (27). The reference values from the Brazilian equations were used (16).

### Statistical analysis

The Shapiro-Wilk test was applied to verify the hypothesis of normality of the measurements. The patients were categorized into 2 groups according to the SIII_N2_ (SIII_N2_ ≤120% or SIII_N2_ >120%) (24). Comparisons between the 2 groups according to demographic variables, clinical variables, cardiopulmonary function, CT, and 6-min walking distance (6MWD) results were performed with the Mann-Whitney test for numeric data and the chi-square or Fisher's exact test for categorical data. To evaluate the association of the SIII_N2_ with all other variables, the Spearman correlation coefficient (r_s_) was used for numerical data and the Mann-Whitney test for 2 subgroups or the Kruskal-Wallis ANOVA followed by the Dunn multiple comparison for 3 subgroups. Data analysis was performed using SAS 6.11 software (SAS Institute, Inc., USA). The significance criterion adopted was of 5%.

## Results

Among the 47 patients evaluated for inclusion in the study, 9 were excluded for the following reasons: VOC in the last 4 weeks (n=3), blood transfusion in the last 3 months (n=2), reported smoking >10 packs per year (n=2), ACS in the last 4 weeks (n=1), and history of pleural tuberculosis (n=1). Thus, the evaluated sample consisted of 19 men and 19 women with a median age of 28 (20–38.3) years. The median Hb concentration was 7.80 (7.57–8.83) g/dL. A TRV >2.5 m/s was observed in 42.1% of the patients. In the CT scans, 13.2% of the patients had no abnormalities, whereas 44.7 and 42.1% had a predominance of reticular and GGO patterns, respectively; however, these CT abnormalities were discrete in the vast majority of cases. Demographic, clinical, echocardiographic, and CT data according to the SIII_N2_ values are shown in Table 1.

**Table 1.** Demographic, clinical, echocardiography and computed tomography data according to phase III slope of the nitrogen single-breath washout test (SIII_N2_).

Variables	Patients with SIII_N2_ ≤120% (n=17)	Patients with SIII_N2_ >120% (n=21)	P value
Demographic data			
Gender (male)	9 (52.9%)	10 (47.6%)	0.74
Age (years)	26 (22–35)	28 (19–41.5)	0.86
Body mass (kg)	63 (53.8–67)	60 (52–67)	0.60
Body height (cm)	165 (161–172)	164 (155–174)	0.64
BMI (kg/m^2^)	22 (20.4–24.5)	21 (19.6–23.7)	0.37
Clinical characteristics			
Hemoglobin (g/dL)	8.20 (7.72–9.08)	7.62 (7.43–8.64)	**0.048**
Frequency of VOC			
≤1	12 (70.6%)	13 (61.9%)	0.57
≥2	5 (29.4%)	8 (38.1%)	
History of ACS	4 (23.5%)	16 (76.2%)	**0.001**
Hydroxyurea therapy	12 (70.6%)	12 (57.1%)	0.39
Echocardiography			
Ejection fraction (%)	70.1 (63.7–74.7)	69.5 (62.9–73)	0.60
TRV (m/s)	2.23 (1.73–2.59)	2.72 (2.09–2.94)	**0.039**
Computed tomography			
Normal	5 (29.4%)	0 (0%)	**0.002**
GGO predominance	9 (52.9%)	7 (33.3%)	
Reticular pattern predominance	3 (17.6%)	14 (66.7%)	

Results are reported as median (interquartile ranges) or number (%). BMI: body mass index; VOC: vaso-occlusive crisis; ACS: acute chest syndrome; TRV: tricuspid regurgitation velocity; GGO: ground-glass opacification. Statistical analysis was done with the Mann-Whitney test for numeric data and the chi-square or Fisher's exact test for categorical data.

Regarding lung functions, 42.1, 13.2, and 26.3% of the patients had reduced TLC, reduced FEV_1_/FVC, and increased residual volume/TLC (RV/TLC), respectively. In the N_2_SBW test, the SIII_N2_ and CV/VC were >120% in 55.3 and 36.8% of the cases, respectively. In the total sample, the median SIII_N2_ and CV/VC values were 154.5 (102–411) and 90.5 (61–150), respectively. In the walking test, the 6MWD was <80% in 63.1% of the cases. Of the 38 patients, 7 (18.4%) had SpO_2_ <90% after the end of the 6MWT. Compared with baseline, the SpO_2_ decline after the 6MWT was ≥4% in 11 (28.9%) patients. Lung function and functional capacity parameters according to the SIII_N2_ values are reported in Table 2.

**Table 2.** Lung function and functional capacity data according to phase III slope of the nitrogen single-breath washout test.

Variables	Patients with SIII_N2_ ≤120% (n=17)	Patients with SIII_N2_ >120% (n=21)	P value
Lung function			
FVC (% predicted)	87 (80–96.5)	68 (63–77)	**<0.0001**
FEV_1_ (% predicted)	88 (75.5–94)	65 (57–75)	**0.001**
FEV_1_/FVC (%)	81 (78–85)	80 (73.5–83)	0.37
DLco (% predicted)	96.3 (77.6–115)	83.6 (74–98.1)	0.078
FVC/DLco (% reference values)	0.95 (0.84–1.10)	0.86 (0.74–1.14)	0.60
TLC (% predicted)	85 (76.5–96.5)	77 (70.5–82.5)	**0.018**
RV (% predicted)	91 (70–103)	98 (75.5–110)	0.52
RV/TLC (%)	104 (88–112)	107 (96–129)	0.24
MIP (% predicted)	75 (58.5–85)	61 (41–96)	0.40
MEP (% predicted)	57 (43.5–64)	50 (45.5–59.5)	0.40
CV/VC (% predicted)	75 (52.5–140)	100 (65.5–175)	0.23
6-min walk test			
6MWD (% predicted)	77.6 (68.3–100)	62.1 (49.7–77)	**0.002**
ΔSpO_2_ (% Pre-Post 6MWT)	2.45 (1.33–3.48)	3.02 (1.84–4.10)	**0.03**

Results are reported as median (interquartile ranges). SIII_N2_: phase III slope of the nitrogen single-breath washout; FVC: forced vital capacity; FEV_1_: forced expiratory volume in 1 s; DLco: diffusing capacity for carbon monoxide; TLC: total lung capacity; RV: residual volume; MIP: maximal inspiratory pressure; MEP: maximal expiratory pressure; CV/VC: closing volume/vital capacity; 6MWD: 6-min walking distance; SpO_2_: peripheral oxygen saturation. Statistical analysis was done with the Mann-Whitney test.

**Figure 1.**
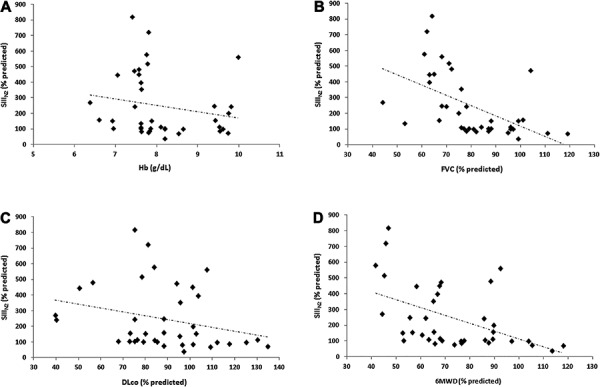
Spearman correlation of phase III slope of the nitrogen single-breath washout test (SIII_N2_) with *A*, hemoglobin (Hb) (r_s_=-0.344; P=0.034); *B*, forced vital capacity (FVC) (r_s_=-0.671; P<0.0001); *C*, diffusing capacity for carbon monoxide (DLco) (r_s_=-0.376; P=0.019); and *D*, the 6-min walking distance (6MWD) (r_s_=-0.554; P=0.0003).

We also evaluated the associations of the SIII_N2_ values with various clinical and laboratory parameters. The correlations of the SIII_N2_ values with the Hb, pulmonary function, echocardiography, and functional capacity data are shown in Table 3 and Figure 1.

**Table 3.** Spearman’s correlation coefficients of phase III slope of the nitrogen single-breath washout test with hemoglobin, lung function, echocardiography and functional capacity parameters of patients with sickle cell anemia.

Variables	SIII_N2_ (% predicted)	
	r_s_	P value
Hemoglobin (g/dL)	−0.344	**0.034**
FVC (% predicted)	−0.671	**<0.0001**
FEV_1_ (% predicted)	−0.636	**<0.0001**
FEV_1_/FVC (%)	−0.110	0.51
DLco (% predicted)	−0.376	**0.019**
FVC/DLco (% reference values)	−0.116	0.49
TLC (% predicted)	−0.440	**0.005**
RV (% predicted)	0.065	0.72
RV/TLC (%)	0.216	0.19
MIP (% predicted)	−0.398	**0.013**
MEP (% predicted)	−0.386	**0.016**
CV/VC (% predicted)	0.280	0.089
Ejection fraction (%)	0.081	0.63
TRV (m/s)	0.316	0.053
6MWD (% predicted)	−0.554	**0.0003**
ΔSpO_2_ (% Pre-Post 6MWT)	0.330	**0.040**

SIII_N2_: phase III slope of the nitrogen single-breath washout; FVC: forced vital capacity; FEV_1_: forced expiratory volume in 1 second; DLco: diffusing capacity for carbon monoxide; TLC: total lung capacity; RV: residual volume; MIP: maximal inspiratory pressure; MEP: maximal expiratory pressure; CV/VC: closing volume/vital capacity; TRV: tricuspid regurgitation velocity; 6MWD: 6-min walking distance; SpO_2_: peripheral oxygen saturation.

## Discussion

The main findings of the present study were that most adults with SCA presented heterogeneity in the ventilation distribution and that a lower frequency of patients presented with small airway disease. A lower serum Hb level, history of ACS, and elevated TRV were associated with ventilation heterogeneity. Moreover, greater heterogeneity in the ventilation distribution led to lower pulmonary volumes, pulmonary diffusion, respiratory muscle weakness, and functional capacity in these patients. Additionally, the inhomogeneity in ventilation increased as the CT changes became more severe.

In the present study, the most common resting lung function finding was elevation of SIII_N2_, which was present in approximately 60% of the evaluated sample. A high SIII_N2_ indicates heterogeneity in the ventilation distribution and reflects differences in the time constants, which are dependent on both lung compliance and airway resistance (28). In SCA, a vaso-occlusive phenomenon may result in pulmonary infarcts, alveolar wall necrosis with airway remodeling, and pulmonary fibrosis with architectural distortion (8,10). The final result of the conjunction of these multiple factors is a loss of pulmonary units that partially contributes to the poor distribution of ventilation (10). Despite the lack of studies on N_2_SBW in SCA, this test has been used for the early diagnosis and stratification of patients and the assessment of the severity of various pulmonary diseases (13,14) and thus may become an important tool to monitor the pulmonary involvement of SCA patients.

Advances in treatment and the survival curves demonstrate that the life expectancy of patients with SCA has increased considerably over the past few decades. The use of hydroxyurea is a determinant contributor that increases the survival of these patients through the reduction of hemolysis and increase in the fetal Hb levels (2,29). However, we found no significant association between the use of hydroxyurea and SIII_N2_ in the evaluated population, which may in part reflect our inability to increase adherence to treatment in patients with SCA who take the drug. We observed an association between the elevation of SIII_N2_ and the ACS report. People with a history of recurrent ACS have a greater possibility of chronic lung injury and development of scarring areas due to pulmonary fibrosis (15,30), which may have a negative impact on the distribution of ventilation. Although ACS has a multifactorial etiology, the main mechanisms implicated in its genesis include pneumonia, fatty embolism, and pulmonary infarction (31); these 3 mechanisms contribute to the poor distribution in ventilation to varying degrees.

Deterioration of lung function is a frequent finding in patients with SCA, mainly among adults (4,8–10). Our findings were similar to those of Ohara et al. (8) and Delclaux et al. (10), who reported that approximately 40% of adults with SCA had restrictive patterns. In SCA, restrictive damage can be explained not only by pulmonary structural changes but also by changes in the thoracic cavity structure due to successive bone infarctions and osteoporosis (8,9). Interestingly, an obstructive pattern has also been described in the PFTs of adults with SCA (32), although this pattern is more common in children (31). In our sample, less than 1/3 of the patients had a reduced FEV_1_/FVC and/or increased RV/TLC; the latter parameter is compatible with air trapping. Although the mechanism is still poorly understood, several hypotheses have been raised to explain the obstructive pattern in patients with SCA, including chronic airway inflammation, bronchial hyper-reactivity, associated asthma, and an increased pulmonary capillary volume (3,33). Interestingly, almost 40% of our population had a high CV/VC ratio in N_2_SBW, which was compatible with small airway disease. Because small airways are the main limiting point for airflow (14), we believe that the obstructive phenomenon is also frequent in adults with SCA and is not detected by the PFTs traditionally used in the assessment of pulmonary function. This finding may have implications in clinical practice because it raises the possibility for controlled and randomized trials with the use of drugs that specifically act at that location in the airway. In our study, the heterogeneity in the ventilation distribution correlated significantly with not only a decrease in pulmonary volumes but also a reduction in the strength of respiratory muscles, which suggests that extrapulmonary mechanisms are also implicated in the worsening of pulmonary ventilation in adults with SCA.

In the CT scans, we observed that more than 85% of the patients had some abnormality (mainly reticular opacities and GGO areas). Our findings were similar to Sylvester et al. (26) and Anthi et al. (34), who also observed a high frequency of reticular and GGO patterns in their patients' CT scans, although the alterations were minimal or mild in most cases. Although a reticular pattern has been attributed to the presence of chronic lesions (especially interstitial fibrosis), the GGO pattern is less specific and may indicate inflammation, partial collapse of the alveoli, an increase in the capillary blood volume, or even fine fibrosis below the resolution of the CT (35,36). Sylvester et al. (26) observed that the FEV_1_, FVC, and TLC correlated significantly with the CT findings, especially the lobar volume loss. A correlation between the extent of interstitial abnormalities in the CT and the number of previous episodes of ACS has also been described (31,37), which suggests that these 2 findings overlap pathophysiologically and may ultimately have repercussions for inhomogeneity in pulmonary ventilation, as observed in the majority of the patients evaluated in our study.

In clinical practice, the 6MWT has been increasingly used for several conditions due to its good reliability and because it reflects the functional level of exercise for activities of daily living (ADLs) (16). Our results were in agreement with those of other investigators, who also noted a decrease in the 6MWD in patients with SCA (4,8). Some mechanisms have been implicated in the poor performance of patients with SCA during the 6MWT, including a low Hb level, deterioration of pulmonary function, low red blood cell deformity, presence of ACS or silent infarction, and high TRV (4,17,18). The low oxygen transport capacity seems to be the most important cause for the reduction in the exercise capacity in SCA patients because several studies have shown a negative association between the 6MWD and the Hb level in these patients (4,17,18). However, Marinho et al. (4) and van Beers et al. (38) showed that a reduced FVC also negatively impacted the functional capacity of SCA patients. Because a strong correlation between SIII_N2_ and FVC was observed in the present study, we hypothesized that these 2 physiological variables reflected a similar pulmonary structural damage phenomenon, which in turn led to a lower tolerance for exercise in adults with SCA. In line with our findings, Di Liberto et al. (39) also observed oxygen desaturation during the 6MWT in some adults with SCA, which was explained by their high percentage of dense red blood cells. In our study, a significant correlation between SIII_N2_ and ΔSpO_2_ was demonstrated, which suggests that a poor distribution in the ventilation is also involved in the oxygen desaturation during the 6MWT in adults with SCA. Interestingly, Liem et al. (15) demonstrated that the inefficiency of pulmonary ventilation detected in cardiopulmonary exercise testing (CPET) was a determinant factor for the decrease in the exercise capacity in children and young adults with SCA, thus highlighting an important role for the ventilatory capacity in these patients during the exercise.

Some limitations of this study should be highlighted. First, the absence of a control group can make data analysis more difficult; however, the pulmonary function and functional capacity data were interpreted by taking into account the predicted values and therefore were corrected for anthropometric data, such as age, gender, height, and body mass. Second, the assessment of the extent of abnormalities in the CT scans could have contributed to a more detailed correlational study with the N_2_SBW test, although almost all of our patients had minimal or minor lesions in the CT scans. Third, the CPET could provide more robust data concerning the type and severity of the exercise limitation in our SCA patients because this method is considered the gold standard for assessments of exercise performance. However, 6MWT better mimics the ADLs because it is a submaximal test that is easy to perform and has no risk for VOC in SCA patients (4,5). Because there has been a significant improvement in the survival of patients with SCA over recent decades (40), we believe that the N_2_SBW test may be a complementary tool to traditional PFTs, especially for the early diagnosis of pulmonary involvement, which tends to become more frequent with aging in this patient population.

In conclusion, the present study shows that heterogeneity in the ventilation distribution is one of the most common pulmonary dysfunctions in adults with SCA. Furthermore, a relationship was found between poor ventilation distribution, worsening pulmonary structural damage, and a lower performance during exercise. Thus, we believe that SIII_N2_ has the potential to become a biomarker of lung function damage in the future and a predictor of exercise intolerance in adults with SCA.
